# Optimization and Evaluation of *Cannabis*-Based Magistral Formulations: A Path to Personalized Therapy

**DOI:** 10.3390/ph18010073

**Published:** 2025-01-09

**Authors:** Bożena Grimling, Magdalena Fast, Magdalena Okoniewska, Artur Owczarek, Bożena Karolewicz

**Affiliations:** 1 Department of Drug Form Technology, Wroclaw Medical University, Borowska 211 A, 50-556 Wroclaw, Poland; bozena.grimling@umw.edu.pl (B.G.); magdalena.fast@umw.edu.pl (M.F.); 2 Pharmaceutical Company Okoniewscy “Vetos-Farma” Sp. z o.o., ul. Dzierżoniowska 21, 58-260 Bielawa, Poland; magdalena.okoniewska@vetos-farma.com.pl

**Keywords:** *Cannabis extractum normatum*, MCT oil, stability study, vaginal drug forms, suppository base, THC, CBN, CBD, endometriosis treatment

## Abstract

Introduction: The official implementation of pharmaceutical-grade cannabis raw materials for medicinal use has permitted doctors to prescribe and pharmacists to prepare cannabis-based formulations. The objective of the pharmaceutical development and manufacturing process optimization work was to propose a suppository formulation containing doses of 25 mg and 50 mg of tetra-hydrocannabinol (∆-9-THC) as an alternative to existing inhalable or orally administered formulations. The formulation could be used for rectal or vaginal administration, thereby providing dosage control in the treatment of endometriosis and other conditions involving pain. In this study, two substrates from suppositories with standardized *Cannabis extractum normatum* (CEX) were used: cocoa butter and Witepsol^®^ H15. Materials and Methods: The long-term stability of CEX was investigated over a period of up to 24 months. The concentrations of ∆-9-THC, cannabidiol (CBD), and cannabinol (CBN) were determined using an HPLC method. Furthermore, the water content of the extract, the ethanol residue, and the microbiological purity were determined. The pharmaceutical properties of CEX-incorporated suppositories, namely content uniformity, hardness, softening time, total deformation time, disintegration time, and the release profile of ∆-9-THC, CBD, and CBN, were evaluated in order to develop optimal preparation procedures for pharmacists. Results and Discussion: Following a 24-month stability study on CEX, no significant alterations in component content were observed beyond the specified requirements. The disintegration time, total deformation time, and hardness of the suppositories based on Witepsol^®^ H15 with CEX were found to be longer and higher, respectively, than those of suppositories formulated with cocoa butter. In vitro studies demonstrated that suppositories prepared with Witepsol^®^ H15 exhibited superior release of ∆-9-THC compared to those prepared with cocoa butter. Conclusions: We suggest that pharmacists making prescription drugs in a pharmacy setting in the form of medical marijuana suppositories will receive a better release profile of the drug by choosing Witepsol® H15 as a substrate.

## 1. Introduction

Knowing properties in prescription preparations is crucial to ensure the safety and efficacy of treatment, especially if the source of active components is a pharmaceutical raw material of plant origin or if its extracts belong to the category of narcotic drugs [1]. Despite the approval in many countries of the raw material for formulation in the form of the dried tops of flowering female shoots of *Cannabis sativa* called *Cannabis flos*, there is a clinical need for forms of the prescription drug other than the dried form for vaporization, or oral forms, i.e., oil extracts, that contain ∆-9-tetrahydrocannabinol (THC) in their composition [2]. In the United States, the proportion of patients hospitalized with chronic pain who reported using cannabis doubled between 2011 and 2015; next, between 2016 and 2020, enrollment in medical cannabis programs increased 4.5-fold [3,4]. In Canada, a large-scale survey of patients living with chronic pain revealed that the prevalence of cannabis use for pain management was 30.1%. Surveys of patients with cancer conducted in the US, Canada, and Europe reported use between 13% and 70% of patients. Registry in Israel includes over 10,000 patients with chronic medical conditions, including cancer, many of whom are elderly and using a suite of medical cannabis preparations. Finally, the majority of these patients take more than one form, and frequency of use varies [5,6,7]. In Australia, prescribed medical cannabis is consumed predominately by oral routes (72%) [8]. Solid dosage forms, i.e., suppositories or globules containing preparations obtained from *Cannabis flos* can be a topical form of the drug, which allows direct action on the areas affected by inflammation, with single-dose forms for rectal or vaginal administration providing dosage control in the treatment of gynecological endometriosis-related pain, myofascial pelvic pain, pelvic congestion syndrome, pelvic inflammatory disease, adenomyosis, hydrosalpinx, and other conditions involving pain, viz, hemorrhoids, rectal fissure, interstitial cystitis/bladder pain syndrome (IC/BPS), Crohn’s disease, ulcerative colitis, and other inflammatory conditions [9,10]. The incidence of endometriosis alone is estimated at 5% to 11% of women of reproductive age and 2% to 5% of cases in postmenopausal women [11]. Chronic pelvic pain associated with endometriosis (CPP) refers to a variety of painful symptoms, including painful menstruation, dyspareunia (pain during sexual intercourse), fatigue, dyschezia (pain during bowel movements), and pain during urination [11,12,13,14,15]. In addition, endometriosis patients are often diagnosed with various comorbidities, including irritable bowel syndrome, rheumatoid arthritis, psoriasis, anxiety, depression, and chronic fatigue syndrome [12,16,17]. Recent studies suggest that endometriosis patients have dysfunction of the endocannabinoid system (ECS) [18], indicating the need for targeted pain management through the modulation of the aforementioned ECS [19]. Available treatments for endometriosis are considered suboptimal, often pointing to the lack of efficacy and the presence of side effects of many of the drugs used in therapy [20]. Typical treatments include use of non-opioid and opioid analgesics, hormone therapy, and anti-neuropathic drugs. Opioid analgesics are not recommended for CPP due to both a lack of efficacy and safety concerns regarding chronic use, often in combination with benzodiazepines [12,21]. The combination used poses a significant risk of cognitive impairment, addiction, and severe withdrawal symptoms [12].

The aforementioned myofascial pelvic pain (MPP) affects 22–94% of patients with chronic pelvic pain and is characterized by the presence of painful trigger points in the pelvic floor muscles and connective tissue and is usually associated with allodynia and hyperalgesia. Myofascial tenderness is a common feature of chronic pelvic pain and is a significant source of discomfort for patients. MPP is often refractory to common treatments due to its coexisting musculo-fascial component, central sensitization, and chronic inflammation [21]. Treatment of MPP is difficult and often requires a combination of synergistic therapies and includes nonsteroidal anti-inflammatory drugs, muscle relaxants, neuromodulators, and pelvic floor physical therapy [22]. Yang et al. used descriptive analyses and collected information on pelvic pain history, cannabis use status, preference for cannabis use, verified opioid abuse risk assessment, and interest in using gynecological *Cannabis flos* products, indicating their potential in therapy.

Anal fissure (AF) is a painful condition characterized by a linear rupture or tear of the squamous epithelium of the lower half of the anal canal. It can occur at any age, affecting men and women equally. Additionally, AF can also develop in people with Crohn’s disease, sexually transmitted diseases, tuberculosis, local postoperative trauma, anal cancer or chemotherapy. Both acute AF lasting less than 2 months and chronic AF lasting more than 6–8 weeks are associated with rectal pain, cramping, and/or bleeding during defecation. Currently, the initial treatment of AF consists of topical preparations with calcium channel blockers, nitroglycerin, and laxatives and topical anesthetic creams [23]. They have gained popularity mainly because they allow for early intervention, allowing for symptom relief by reducing the contraction and spasm of the rectal muscles, which reduces the risk of re-injury during defecation.

The search for new pain treatment options is therefore considered urgent research and a clinical priority for the treatment of endometriosis, anal fissure, and myofascial pelvic pain, among others [16]. Recent surveys show that nearly nine out of ten women with endometriosis are willing to consider participating in a clinical trial of medical marijuana for the treatment of chronic pelvic pain, indicating the need to develop new therapeutic approaches [21]. The legalization of cannabis in Canada in 2012 increased its use by 8.2% in patients with chronic pelvic pain, while one in five patients have been shown to use *Cannabis flos* products for pain relief [24]. In addition, more than half of the patients who had never used marijuana expressed a desire to use it to treat pelvic pain [21]. Rectal and vaginal forms of medical marijuana containing 10 mg of ∆-9-THC available in the commercial formulation of Rectal THC Medical Cannabis (Panaxia Pharmaceutical Industries Ltd., Lod, Israel) suppositories and Vaginal THC Medical Cannabis (Panaxia Pharmaceutical Industries Ltd., Lod, Israel) suppositories are recommended for relieving pain, i.e., neuropathy/chronic/stiffness, reducing nausea, increasing appetite, relieving spastic conditions, and painful spasms [25]. Due to the growing interest and use of parenteral forms from *Cannabis flos*, this study used a ready-made standardized *Cannabis extractum normatum*, an oil extract registered in Poland as a pharmaceutical raw material, to prepare globules/suppositories under pharmacy formulation conditions. In the present study, the stability of *Cannabis extractum normatum*, including, i.e., ∆-9-THC, CBD, and CBN contents, water content, ethanol residue, and microbiological purity, in 24 months was evaluated, and technology for preparing cocoa butter-based and Witepsol-based suppositories with extractum was proposed. The pharmaceutical properties of the suppositories, namely content homogeneity, hardness, softening time, total strain time, disintegration time, and the release profiles of ∆-9-THC, CBD, and CBN, were evaluated to develop optimal preparation procedures for pharmacists. Optimizing fatty-based suppository formulation with cannabis oil and assessing their properties, including the pharmaceutical availability of ∆-9-THC, CBD, and CBN from the formulations, will allow a comprehensive evaluation and extension of the available therapeutic options. Table 1 illustrates the prescriptions for suppositories containing cannabis extract that were issued by doctors in Poland and subsequently filled at a pharmacy.

## 2. Results and Discussion

### 2.1. Stability Test of Cannabis Extractum Normatum

Stability studies constitute an indispensable element of the pharmaceutical development process, facilitating a critical assessment of the therapeutic potential of an active pharmaceutical ingredient (API) or a final pharmaceutical product under the influence of diverse environmental factors [26]. It is well established that the potency of cannabis products, including cannabis extractum, is dependent on the cannabinoid profile, including the Δ9-tetrahydrocannabinol (THC) content [27]. The psychoactive cannabinoid Δ9-THC exhibits differential stability in various environmental conditions. Lindholst [28] demonstrated that the cannabinoid stability in cannabis products, is influenced by light, temperature, and oxygen availability. Furthermore, it has been demonstrated that the potency of herbal cannabis decreases as its storage time increases. The daylight and normal temperatures accelerate the degradation processes of both Δ9-THC and CBD (cannabidinol), thereby increasing the formation degree of CBN (cannabinol) [29]. While the degradation of Δ9-THC to CBN appears to be of a more chemical nature, the degradation of CBD appears to be of a more biochemical nature because the possible degradation route is cyclization-catalyzed by CBD-cyclase [30]. The experimental results regarding the stability of the major cannabinoids highlighted a small but constant difference between cannabinoids content of *Cannabis extractum* in storage conditions. After 24 months of conducting the stability study, no important changes were recorded in components content beyond the specification requirements (see Table 2). A slight decrease in THC content was observed in the extract from 103.65 mg/g at the initial time to 98.76 mg/g after 24 months. Similar results were observed for CBD content decreasing from 3.37 mg/g to 2.76 mg/g at the last point of the stability test. Cannabinol (CBN) is the product of degradation, or oxidation, of tetrahydrocannabinol (THC). When THC is exposed to oxygen and heat, over time, it breaks down to CBN. The values of the labeled CBN remained within the range of 0.03 mg/g throughout the duration of the test. No changes in the microbiological purity of the *Cannabis extractum normatum* samples were observed.

### 2.2. Physicochemical Characteristics of Suppository

**Figure 1:**
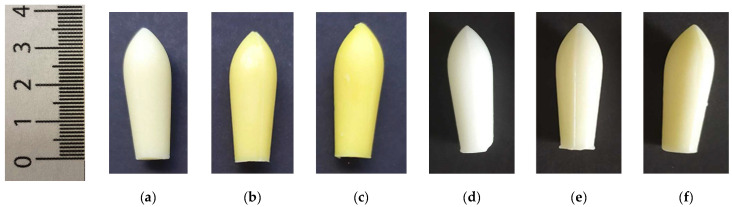
Appearances of suppository formulations with *Cannabis extractum normatum* prepared based on cocoa butter and Witepsol^®^ H15: (**a**) CB; (**b**) CB_EXT_25; (**c**) CB_EXT_50; (**d**) W; (**e**) W_EXT_25; (**f**) W_EXT_50.

The model suppositories were torpedo-shaped, smooth in texture, and free of entrapped air, contraction holes, and brittle fracture. Upon visual examination with the naked eye and a hand lens, the external and internal surfaces of the suppositories displayed uniformity in appearance. This uniformity was observed in terms of color and texture. All prepared suppositories had a shiny, smooth surface, torpedo shape, and smooth texture, without trapped air. The appearances of the suppositories prepared from cocoa oleum and Witepsol^®^ H15 with MCT oil and with *Cannabis extractum normatum* on an MCT oil base are shown in Figure 1a–c and Figure 1d–f, respectively.

The obtained suppositories formulations exhibited uniformity of mass, as illustrated in Table 3. This indicates that the oil was adequately dispersed and that all the batches met the standards set out in the European Pharmacopoeia. The standard deviations were low for all the batches, indicating that the mixture of ingredients and the suppository base were relatively homogeneous prior to pouring into molds. The average weight for all the formulations was within the Pharmacopoeia limit. Most standard deviations did not exceed 1.00%, representing a well-calibrated mold. All suppositories on lipophilic bases were deformed within 15 min. The softening time of lipophilic suppositories was observed to decrease with an increase in the percentage of *Cannabis extractum normatum* addition. The addition of 0.3 g/suppository of *Cannabis extractum normatum* into the Witepsol-base suppository formulation increased the softening time to 9.50 min, whereas this time for the series obtained with 0.6 g/suppository of added oil was 5.62 min. Suppositories based on cocoa with 0.6 g/suppository exhibited a softening time of 3.49 min. Similarly, the hardness of the suppositories was affected by the addition of oil extract, causing a decrease in hardness as the proportion of oil in the formulation increased. The objective of this test was to evaluate the strength of the suppositories and to ascertain whether the prepared formulation could withstand the risks associated with packaging and shipping. According to European Pharmacopoeia, the decomposition time of the suppository should not exceed 30 min for formulation on the lipophilic base. The prepared vaginal suppository formulation showed a decomposition time between 4.12 ± 0.34 min and 9.20 ± 1.09 min. The results follow Pharmacopoeia requirements [26]. Table 4 lists the mean contents of ∆-9-THC, CBD, and CBN in the suppositories, respectively. The THC content of the suppository series ranged from 83.04 to 89.9% of the declared content.

### 2.3. Release Test Study from Fatty Suppository Bases

**Figure 2:**
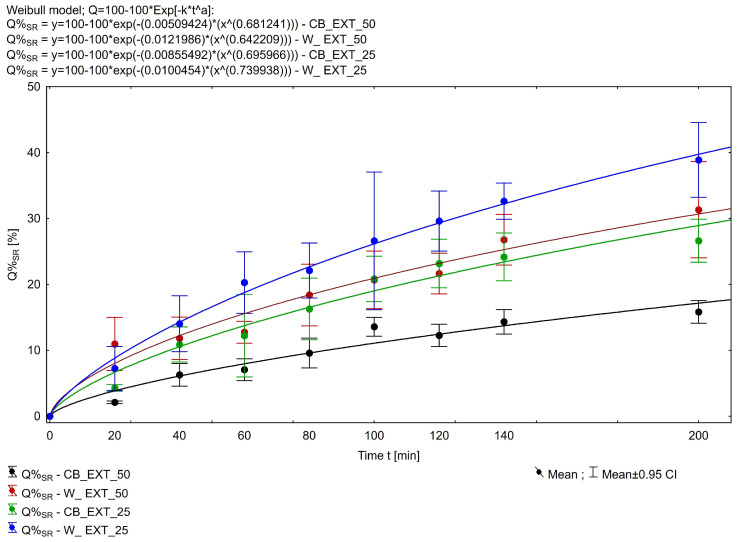
In vitro ∆-9-THC release profiles from suppositories. Each data point represents mean ± SD, n = 3.

The phytocannabinoids (THC and CBD) are categorized as Biopharmaceutics Classification System class II drugs with high lipophilicity (logP of ~6.3 and ~6.97, respectively), low water solubility (12.6 and 28.0 mg/L, respectively), and a pKa of 9.29 and 10.6, respectively [31]. To evaluate the release of ∆-9-THC, CBD, and CBN from the suppositories, the phosphate buffer (pH = 7.4) with the addition of 2.0% sodium lauryl sulfate was used. The release of THC from Witepsol^®^ H15-based suppositories was more efficient than from those based on cocoa butter; more than 38% of the drug released within 200 min from the W_EXT_25 formulation, while more than 26% of THC was released in this time from the CB_EXT_25 formulation. The release of THC from batches W_EXT_50 and CB_EXT_50 containing 50 mg of THC was lower and resulted in >31% and >15% released, respectively. Figure 2 shows the release profile of THC from the prepared fatty suppository bases. Dissolution efficiency (DE), the area under a dissolution curve between defined time points, was compared for the characterization of dissolution profiles. Determined for the four compared formulations, the mean values of the THC dissolution efficiency (Equations (1)–(3)) were compared with each other using parametric one-way ANOVA. The LSD post hoc test was used for multiple comparisons of each type. The normality of the distributions of the compared variables was assessed with the W Shapiro–Wilk test, and the homogeneity of variance, with Levene’s and Brown–Forsythe tests. The analyzed variables met the criterion of normality and homogeneity of variance. The significance level employed in all statistical analyses was α = 0.05. Dissolution efficiency (DE) is define as in Equation (1):

(1) $$DE=\frac{AUC}{y_{100\%}\cdot t}=\frac{\int_{0}^{t}Q\left(t\right)dt}{1\left[100\%\right]\cdot t}$$

where $Q\left(t\right)$ is the percentage/fraction of drug released from the drug formulation after time, and x was determined with the classical method of calculating *AUC* by summing the trapezoidal areas according to Equation (2).

(2) $$\int_{0}^{t}Q\left(t\right)dt=\int_{0}^{t_{n}}Q_{n}\left(t\right)dt=\sum_{i=1}^{n}\frac{Q_{i+1}\left(t\right)+Q_{i}\left(t\right)}{2}\cdot \left(t_{i+1}-t_{i}\right)$$

Finally,

(3) $$DE=\frac{\sum_{i=1}^{n}\frac{Q_{i+1}\left(t\right)+Q_{i}\left(t\right)}{2}\cdot \left(t_{i+1}-t_{i}\right)}{1\cdot t_{last}}$$

**Figure 3:**
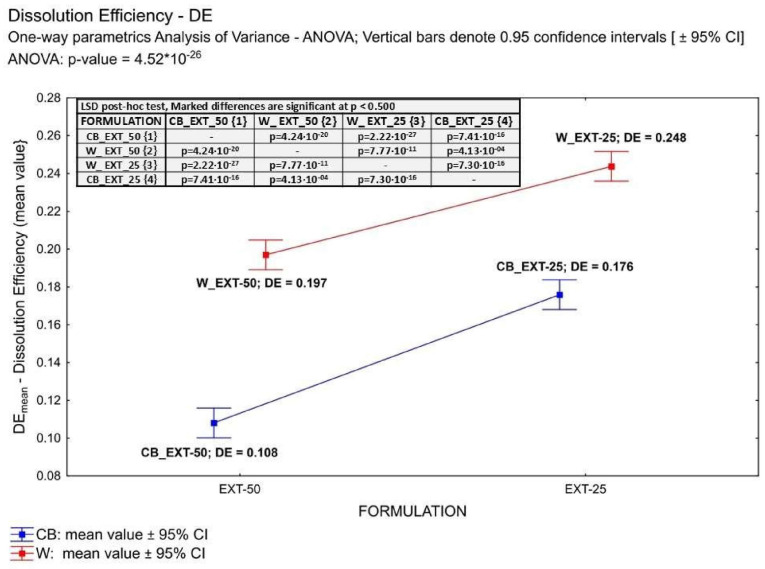
Comparing THC dissolution efficiency (DE) from suppository formulations using parametric one-way ANOVA.

**Figure 4:**
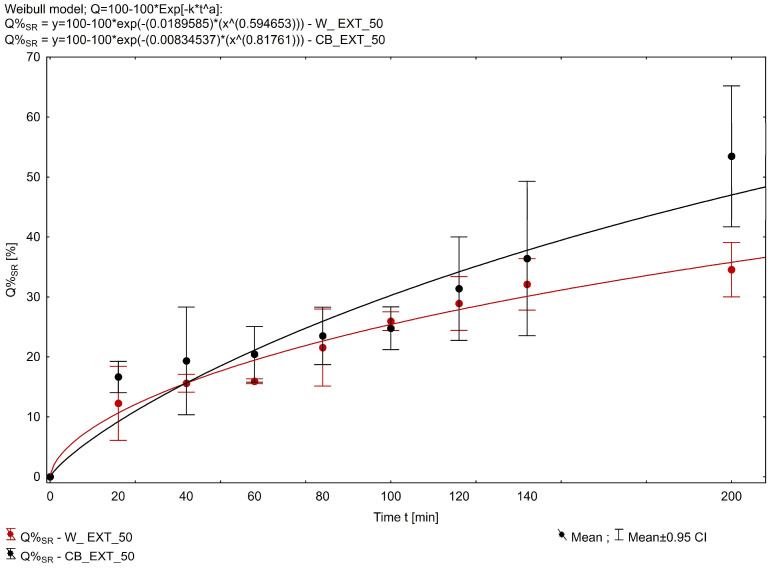
In vitro CBD release profiles from suppositories. Each data point represents mean ± SD, n = 3.

**Figure 5:**
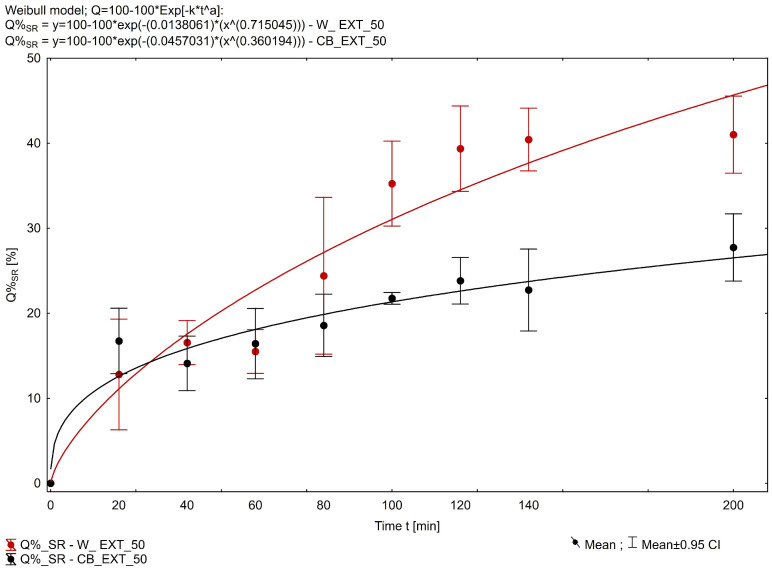
In vitro CBN release profiles from suppositories. Each data point represents mean ± SD, n = 3.

The empirical parameter DE was found highest for the Witepsol-based formulations (Figure 3), and the DE values for W_EXT-50 and CB_EXT-50 were 0.197 and 0.108, respectively. In contrast, for the W_EXT-25 and CB_EXT-25 formulations, the DE values were 0.248 and 0.176, respectively. The cumulative amounts of CBD and CBN released from the suppository formulation containing 0.6 g of *Cannabis extractum normatum* (50 mg of THC/suppository) versus the time are presented in Figure 4 and Figure 5. More than 50% of the loaded CBD was released from the CB_EXT_0.5 formulation in the first 200 min, while less than 35% of CBD was released from the W_EXT_0.5 formulation at the same time. In the case of CBN analysis, more than 40% of the loaded substrate was released from the W_EXT_50 formulation within the first 200 min, while less than 30% was released from the CB_EXT_50 formulation, respectively, within the same period time.

To the authors’ knowledge, that this is the first study in Poland to assess the quality of a magistral suppository prepared in a community pharmacy setting, comprising THC and CBD, and to examine the pharmaceutical stability of these substances. Overall, the results obtained in this investigation demonstrate that the technology for the preparation of suppository oily *Cannabis extractum* content had a significant impact on their organoleptic characteristics. As a result of placing suppositories in the refrigerator too quickly, changes in appearance in the form of voids were noticeable. This phenomenon is described in the literature as unfavorable [32]. The presence of voids promoted the crushing of the suppository, for example, when removing it from the mold. During the studies described in this paper, an increase in the solidification time of suppositories containing *Cannabis extractum normatum* was noted compared to suppositories prepared from the substrate alone. This observation applies to suppositories prepared with cocoa butter as well as with Witepsol^®^ H15. The pharmacist preparing the drug should maintain a longer time interval between pouring the mass into the molds and placing the suppositories in the refrigerator compared to suppositories containing no extract. All tested formulations met pharmacopoeial requirements for mass uniformity, and the maximum deviation from the average mass was 1.65%. The active components’ release rate indicates their sustained release potential. In considering the release profile and medicinal cannabis potential in the form of magistral formulations, these results represent valuable data for physicians using this therapy option as an adjuvant therapeutic intervention in the management of chronic pain and associated comorbidities [33]. Ramella et al., in their studies, described that the MCT lipid source may be an important lipid for use in the formulation of medical cannabis-based oils [34]. We are aware that our study was subject to certain methodological limitations, particularly the lack of available comparative research in this area. Further investigation into the effect of varying suppository compositions on the release of THC and CBD and the subsequent impact on the physicochemical properties and bioavailability of the active ingredients would enable a more comprehensive understanding of the role of suppository substrates in determining the efficacy of hemp oil bus formulations. In our study, we successfully demonstrated, after 24 months of a stability study, that in the *Cannabis extractum normatum* prepared based on MCT oil, no changes in the content of the phytocannabinoid constituents beyond the specification requirements were observed. The choice of oil for the preparation of the extract and suppository base, together with obtained results of release profile of the active substances, help in next step will allow to evaluation of real-practice release experience with clinical observation results.

## 3. Materials and Methods

### 3.1. Materials

*Cannabis extractum normatum* available in base medium-chain triglyceride oil (EXT) ∆-9-THC 10%, CBD < 1% (Permit number 30067, Serial number 02012021) was received as a donation from the Pharmaceutical Company Okoniewscy “Vetos-Farma” Sp. z o.o. (Bielawa, Poland). Cocoa butter (CB) was provided by Fagron Pharma Cosmetic (Kraków, Poland). Medium-chain triglyceride (MCT oil) was provided by Gustav Heess Sp. z o.o. (Warsaw, Poland). Sodium lauryl sulfate (SLS) and phosphoric acid were purchased from Sigma-Aldrich Chemical Company (St. Louis, MO, USA). Witepsol^®^ H15 (W) was provided by Actifarm Sp. z o.o. (Krakow, Poland). Hydrochloric acid 35–38% and absolute ethanol ≤99.8% were purchased from P. P. H. Stanlab (Lublin, Poland). HPLC-grade acetonitrile and phosphate buffer concentrate, pH 7.4, were provided from Chempur (Piekary Slaskie, Poland). Δ9-Tetrahydrocannabinol (∆-9-THC) reference standard of 1.0 mg/mL in 1 mL methanol was purchased from Lipomed Inc., (Cambridge, MA, USA). Cannabidiol (CBD) and cannabinol (CBN) reference standards were purchased from Lipomed Inc. (Cambridge, MA, USA). All chemicals used in this study were of analytical grade.

### 3.2. Long-Term Stability Study of Cannabis Extractum Normatum

The *Cannabis extractum normatum* prepared on medium-chain triglyceride oil base was kept in 10 mL closed amber glass bottles with plastic caps at room temperature (25  ±  2 °C) and 60%  ± 5 RH for 24 months within stability chambers. At each time point after 3, 6, 9, 12, and 24 months, the visual appearance of the samples was controlled, and the ∆-9-THC, CBD, and CBN contents were determined using the HPLC method. In addition, the moisture content in the extract, ethanol residue, and microbiological purity were determined.

#### 3.2.1. Determination of the Total Water Content

Karl Fischer titrations were performed in accordance with European Pharmacopoeia (Eur. Ph.) monograph 2.5.12. [26]. The instrument used was a Mettler Toledo DL50 Graphix (Greifensee, Switzerland) calibrated with a predetermined mass of water (25–30 μL) and a Hydranal^®^ water standard 10.0 (1 g contains 10.0 mg water = 1.0%). Approximately 200 mg of each sample was used for the moisture determination.

#### 3.2.2. Determination of Residual Ethanol

Residual ethanol was determined according to the European Pharmacopoeia recommendations (chapter 2.4.24.) [26]. The method was implemented on a gas chromatographic system (GC) (Agilent Technologies, Santa Clara, CA, USA, model 7890B) coupled to a headspace sampler (Agilent Technologies, Santa Clara, CA, USA, model 7697A) and a mass selective detector (Agilent Technologies, Santa Clara, CA, USA, model 5977A). Standards and samples solutions were prepared in dimethyl sulfoxide. After the incubation of the sample (5 mL in a 10 mL headspace vial) at 120 °C for 15 min, during which it was shaken, 0.5 mL of the vapor phase was injected into the GC/MS system in split injection mode (split ration 6.8:1). The temperatures of the headspace loop, the transfer line, and the EPC volatiles interface were 135 °C, 145 °C, and 160 °C, respectively. The solvents were separated on a Zebron ZB 624 capillary column (60 m × 0.32 mm, 1.8 μm film thickness) (Phenomenex, Torrance, CA, USA). The oven temperature was programmed at 25 °C/min from 60 °C (initially held for 5 min) to 270 °C (finally held for 10 min). The total run time was 23.4 min. The temperatures of the injection port, the ion source, the quadrupole, and the interface were set at 160 °C, 230 °C, 150 °C, and 280 °C, respectively.

#### 3.2.3. Microbiological Purity Test

The microbiological purity test was carried out in accordance with European Pharmacopoeia monograph 2.6.12. The microbiological examination of non-sterile products was as follows: Microbial enumeration tests were carried out in accordance with monograph 2.6.13 [26]. The microbial contamination of the solution was evaluated using culture-dependent methods in compliance with the quality parameters for herbal medicinal products set out in the European Pharmacopoeia 11th Edition (Council of Europe, 2023) [26]. This provides a detailed description of the parameters, protocols, and materials to be used in the microbial analysis. In particular, the total aerobic microbial count (TAMC), the total combined yeasts/mold count (TYMC), and the semiquantitative estimation (order of magnitude) of bile-tolerant Gram-negative bacteria (BTGNB) were determined and expressed as colony-forming units (CFU) per gram. The absence of Escherichia coli and Salmonella spp. was also assessed. Under sterile conditions, precisely 10 g of product was measured into a glass flask containing 5 g of Tween 80 and sterile pearls for shaking. Then, 85 mL of sterile water was added and mixed thoroughly.

##### Total Aerobic Microbial Count (TAMC) Determination

After filtrating 10 mL of the solution obtained previously, using a sterile membrane filter with a 0.45 µm pore size, and after washing with 3 portions of 100 mL of pH 7 buffer, the filter membrane was transferred to a Petri dish with digested casein soya bean digest agar. The membrane was incubated at 30–35 °C for 3–5 days, and then the colonies were counted. The number of CFU per gram or per milliliter of product was calculated.

##### Total Combined Yeast/Mold Count (TYMC) Determination

After filtrating 10 mL of the solution obtained previously, using a sterile membrane filter with a 0.45 µm pore size, and after washing with 3 portions of 100 mL of pH 7 buffer, the filter membrane was transferred to a Petri dish with Sabouraud dextrose agar. The membrane was incubated at 20–25 °C for 5–7 days, and then the colonies were counted. The number of CFU per gram or per milliliter of product was calculated.

##### Test for *Escherichia coli* Absence

After filtrating 10 mL of the solution obtained previously, using a sterile membrane filter with a 0.45 µm pore size, and after washing with 3 portions of 100 mL of pH 7 buffer, the filter membrane was transferred to casein soya bean digest broth. The membrane was incubated at 30–35 °C for 18–24 h, and then, for the selection and subculture, 1 mL of casein soya bean digest broth was transferred to 100 mL of MacConkey broth and incubated at 42–44 °C for 24–48 h. The subculture on a plate of MacConkay agar was incubated for 18–72 h at 30–35 °C. The growth of red, non-mucoid colonies of Gram-negative rods indicated the possible presence of *E. coli.*

##### Test for Salmonella Absence

After filtrating 50 mL of the solution obtained previously, using a sterile membrane filter with a 0.45 µm pore size, and after washing with 3 portions of 100 mL of pH 7 buffer, the filter membrane was transferred to casein soya bean digest broth. The membrane was incubated at 30–35 °C for 18–24 h, and then for the selection and subculture, 0.1 mL of casein soya bean digest broth was transferred to 10 mL of Rappaport Vassiliadis Salmonella enrichment broth and incubate at 30–35 °C for 18–24 h. A subculture from Rappaport Vassiliadis broth was sifted on plates of xylose, lysine, and deoxycholate agar and incubated at 30–35 °C for 18–48 h. The possible presence of *Salmonella* was indicated by the growth of well-developed, red colonies, with or without black centers.

##### Test for Bile-Tolerant Gram-Negative Bacteria

After filtrating 10 mL of the solution obtained previously, using a sterile membrane filter with a 0.45 µm pore size, and after washing with 3 portions of 100 mL of pH 7 buffer, the filter membrane was transferred into 9 mL of casein soya bean digest broth. The obtained broth was incubated at 20–25 °C for a time sufficient to resuscitate the bacteria but not sufficient to encourage the multiplication of the organisms (usually 2 h but not more than 5 h). For isolation and screening, bile-tolerant Gram-negative bacteria were successively added to dilutions of casein and soybean hydrolyzate broth corresponding to 0.1 g, 0.01 g, 0.001 g, and 0.0001 g of the examined product and to the broth-Mossel enrichment for intestinal sticks. To do this, dilutions were carried out from broth with casein and soy hydrolysate, taking 1 mL to 9 mL of saline to a dilution of 0.0001 g of the tested product. Finally, the diluted broth was added to the next tubes with broth-Mossel and incubated for 24–48 h at 30–35 °C. Each dilution culture was tested on a plate of violet red bile glucose agar and incubated for 18–24 h at 30–35 °C. The growth of colonies constituted a positive result and noted the smallest quantity of the product that gives a positive result and the largest quantity that gives a negative result.

### 3.3. Preparation of Suppositories

For the processing of the botanical raw material of *Cannabis flos* and *Cannabis extractum normatum*, this research was authorized by the Chief Pharmaceutical Inspector, permit number NKIS.5510.17.2023.JSO.4 of 8 May 2023.

**Figure 6:**
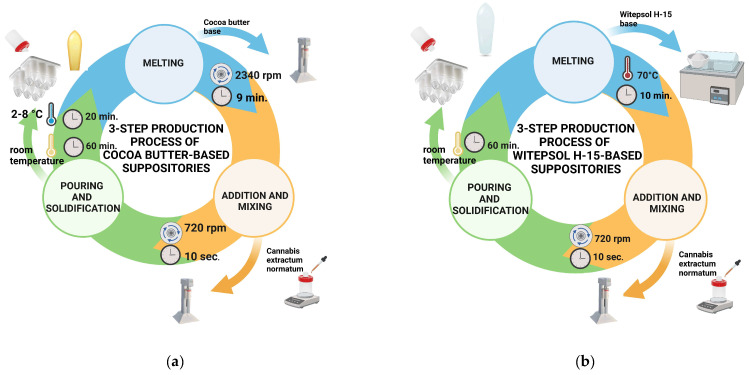
Scheme of technology preparation of suppositories with *Cannabis extractum normatum* on fatty bases: (**a**) cocoa butter; (**b**) Witepsol^®^ H15. Created with Biorender.com.

For this study, suppositories containing raw medium-chain triglyceride oil (placebo series CB_MCT and W_MCT) and *Cannabis extractum normatum* (series CB_EXT and W_EXT) were prepared using a pouring method. A placebo series was initially prepared to design and evaluate suppository preparation technology with raw MCT oil as a component of *Cannabis extractum normatum*. Depending on the type of base, the preparation process was subjected to varying parameters of mixing time and stirrer speed. Stirring started from level 0, which corresponds to 720 rpm in the case of the unguator Eprus^®^ model e/s (Eprus Sp. z o.o., Bielsko-Biala, Poland). The speed was increased every 15 s until it reached level 9, which corresponds to 2340 rpm. The formulation composition and preparing process conditions of the suppositories for each batch are given in Table 5. Eprus 2.0 g plastic suppository molds comprising polyvinyl chloride (Eprus Sp. z o.o., Bielsko-Biala, Poland) were utilized as the final drug form pack. After the suppository mass was poured into molds, the suppositories based on cocoa butter were left at room temperature for 1 h and then stored at either 2–8 °C in the refrigerator for 20 h. In turn, suppositories based on Witepsol^®^ H15, after being poured into molds, were left at room temperature (22 ± 1 °C) for 20 h. After solidification, all series of suppositories were removed from the molds and tested. A scheme of the technology preparation of the suppository placebo series and with *Cannabis extractum normatum* on both fatty bases is presented in Figure 6.

### 3.4. Suppository Analysis

#### 3.4.1. Visual Examination

The suppositories were examined quality control assessments. The visual characteristics of the obtained forms, including their color, shape, and surface texture, were evaluated. The assessment of the surface condition included the presence or absence of the following: luster, dullness, mottling, cracks, axial pits, blisters, and holes.

#### 3.4.2. Mass Uniformity Test of Suppositories

The uniformity of the mass of the single-dose preparations was carried according to the monograph recommendations Ph. Eur. [26]. Twenty suppositories from each series were individually weighted with an electronic scale (XS 205, Mettler Toledo, Columbus, OH, USA). For each suppository series, the average weight and the standard deviation values were calculated.

#### 3.4.3. Disintegration Test for Suppository Forms

A disintegration suppository test was conducted using the Erweka ST32 apparatus (ERWEKA GmbH, Langen, Germany). The apparatus was set to rotate the cylinders 180° every 2 min, and the temperature of the water located in the tank was 36.5 ± 0.5 °C. The study was conducted in triplicate for each suppository series, comprising cocoa butter and Witepsol^®^ H15 with MCT.

#### 3.4.4. Determination of the Softening Time of Lipophilic Rectal Suppositories

The disintegration of suppository test was performed in water maintained at 36.5 ± 0.5 °C using a type B Erweka PM30 apparatus (Erweka GmbH, Langen, Germany). The test was conducted for three suppositories from each formulation. The results of the total deformation time test are presented in Table 3.

#### 3.4.5. Hardness Test

The mechanical strength of the suppositories was determined using a suppository hardness tester Erweka SBT (Erweka GmbH, Langen, Germany) on 10 suppositories at room temperature (22 ± 0.5 °C). The force required to crack each suppository was recorded. Ten suppositories were tested for each prepared series.

#### 3.4.6. Test Study of Suppository Active Substance Release 

The release of ∆-9-THC, CBD, and CBN from the suppositories was investigated using a paddle apparatus (Erweka DT 700, Erweka GmbH, Langen, Germany) containing 400 mL of phosphate buffer (pH = 7.4) with the addition of 2.0% sodium lauryl sulfate at 37 ± 0.5 °C and stirring at 50 rpm. Two-milliliter samples were collected at 20, 40, 60, 80, 100, 120, 140, and 200 min intervals. To maintain the same medium volume, fresh buffer was added at each interval. Subsequently, the samples were filtered through 0.45 μm nylon membrane filters for further HPLC analysis. The released ∆-9-THC, CBD, and CBN were determined by HPLC, in accordance with the methodology described in part 3.4.6.1, for the purpose of determining the ∆-9-THC, CBD, and CBN content in the suppository with the HPLC method.

##### Determination of ∆-9-THC, CBD, and CBN Content in Suppository with HPLC Method

The amount of ∆-9-THC, CBD, and CBN in the suppository series was determined using a Waters Alliance 2695 high-performance liquid chromatograph with Waters 2996 and 2998 PDA Detector. For this purpose, a suppository was dissolved in 200 mL of ethanol, at a temperature of approximately 37 °C, and the next solution was then made up to 250 mL with ethanol in a volumetric flask. Two milliliters of the solution were taken, filtered through a 0.22 μm pore size polytetrafluoroethylene membrane into a chromatography vial, and a 10 μL sample was introduced onto a thermostated ReproShell ODS-1 HPLC column (150 × 3 mm) with a 2.7 μm grain size. The mobile phase A was a phosphoric acid aqueous solution with a concentration of 8.64 g/L, while the mobile phase B was acetonitrile. The flow rate was set at 1.0 mL min^−1^, and elution was conducted in gradient mode, as detailed in Table 6. The separation was conducted at 40 °C with ultraviolet detection at 225 nm, and the injection volume was 10 µL. The THC and CBD contents were calculated based on Equation (4), and the CBN content was calculated based on Equation (5), which were developed by the manufacturer of the extract used in the tests to determine the active substance content of the suppository.

(4) $$X_{THC},X_{CBD}=\frac{P_{\;test.}\times C_{ref.}}{P_{test.}\times m\times 5}\times 1000$$

(5) $$X_{CBN}=\frac{P_{\;test.}\times C_{ref.}}{P_{test.}\times m\times 20}\times 1000$$

The cannabinoid content was calculated in accordance with Equations (4) and (5), respectively, where *X_THC_* represents the THC content [mg/g], *X_CBD_* denotes the CBD content [mg/g], X_CBN_ signifies the CBN content [mg/g], *P_test_*_._ represents the area under the THC/CBD/CBN peak of the test solution, *P_ref_*_._ represents the area under the THC/CBD/CBN peak of the reference substance solution, *C_ref_*_._ represents the concentration of the reference substance [mg/mL], and m is the mass of substance [g]. The concentration of the standard was indicated on the manufacturer’s packaging, thus obviating the necessity for a standard curve.

#### 3.4.7. Statistical Analysis

Results are presented as mean values ± standard deviation, calculated from three measurements unless otherwise specified. In the dissolution study, the mean values of the substance dissolution efficiency from the formulation were compared with each other using a parametric one-way ANOVA test. The LSD post hoc test was used for multiple comparisons of each type. The normality of the distributions of the compared variables was assessed with the W Shapiro–Wilk test and the homogeneity of variance with Levene’s and Brown–Forsythe tests. All the compared variables met the assumptions of normal distribution and homogeneity of variance. For all statistical analyses performed, a significance level of α = 0.05 was assumed. Statistical analysis was performed using STATISTICA PL^®^ version 13.

## 4. Conclusions

The use of cannabis oil formulations in magistral preparations has attracted considerable interest in recent years for two main reasons. Firstly, these preparations offer the ability to develop individualized dosage forms, which is of particular benefit in the context of pain therapy. Secondly, the ease with which the dose can be modulated during treatment is an important advantage [35]. Considering the current lack of pharmaceutical guidelines, evidence accrued through real-practice experience can help inform best pharmaceutical practices in terms of magistral preparation with *cannabis* and their evaluation. This work is supported by the fact that our formulations are well aligned with those reported from prescribing practice in Poland. To evaluate available therapeutic options, we successfully demonstrated that 15% of the THC of the suppositories is released within the first three hours, suggesting that fatty-based semisoft suppositories release THC in a prolonged way, and at the same time, in the clinical effect evaluation of the treatment of endometriosis and other conditions involving pain, the fatty base composition should be considered. The empirical THC dissolution efficiency parameter was found to be highest for Witepsol H15-based suppositories, indicating its potential in cannabis magistral formulations.

## Figures and Tables

**Table 1:** Examples of prescriptions for suppositories containing cannabis extract issued by medical practitioners in Poland.

Suppository Formula with Cannabis Flos Extract			Dose of THC per Suppository/Dosing
Rx. (for 10 suppositories)	10% oil solution with THC from extraction of Cannabis flos THC 22%, CBD 1% (Aurora Deutschland GmbH)	0.25	25 mg/once a day
	Witepsol ^1^	q.s.	
Rx. (for 30 suppositories)	10% oil solution with THC from extraction of Cannabis flos THC 8%, CBD 8% (Aurora Deutschland GmbH)	1.0	10 mg/once a day
	Witepsol ^1^	q.s	
Rx. (for 30 suppositories)	10% oil solution with THC from extraction of Cannabis flos THC 8%, CBD 8% (Aurora Deutschland GmbH)	0.5	5 mg/once a day
	Witepsol ^1^	q.s.	
Rx. (for 60 suppositories)	10% oil solution with THC from extraction of Cannabis flos THC 8%, CBD 7% (Conopy Growth)	12.0	2 × 20 mg per day for an of 5.5-year-old child
	Witepsol ^1^	q.s.	
Rx. (for 30 suppositories)	10% oil solution with THC from extraction of Cannabis flos THC 20%, CBD ≤ 1% (Aurora Deutschland GmbH)	0.01	1 mg per day for an 8-year-old child
	Witepsol ^1^	q.s.	
Rx. (for 30 suppositories)	10% oil solution with THC from extraction of Cannabis flos THC 20%, CBD ≤ 0.5% (Aurora Deutschland GmbH)	0.5	50 mg/once a day for a 57-year-old patient
	Witepsol ^1^	q.s.	

^1^ Witepsol—Witepsol^®^ H15.

**Table 2:** Long-term stability study of *Cannabis extractum normatum* at room temperature (25 ± 2 °C) and 60% ± 5 RH.

Parameter	Specification Requirements	Test Results (Month)					
		0 (Initial)	3	6	9	12	24
Appearance	Light yellow—light brown oily liquid	Light yellow oily liquid	Light yellow oily liquid	Light yellow oily liquid	Light yellow oily liquid	Light yellow oily liquid	Light yellow oily liquid
Assay of ∆-9-THC	90–110 mg/g	103.65 ± 0.8502	95.40 ± 0.8670	94.33 ± 1.3955	95.61 ± 1.0803	91.04 ± 0.0915	98.76 ± 0.0961
Assay of CBD	≤10 mg/g	3.37 ± 0.0188	2.95 ± 0.0309	2.70 ± 0.0299	2.57 ± 0.0510	2.67 ± 0.0137	2.76 ± 0.0455
Purity—CBN content	≤2.5 mg/g	0.03 ± 0.0002	0.03 ± 0.0000	0.03 ± 0.0005	0.03 ± 0.0002	0.03 ± 0.0003	0.03 ± 0.0002
Water	≤0.5% in 0.200 g	0.1465	0.1191	0.2392	0.2464	0.1249	0.1234
Ethanol residue	≤0.5% (5000 ppm)	-	<LOD	<LOD	<LOD	<LOD	<LOD
Microbiological purity	TAMC: ≤10^3^ cfu/g	<1 cfu/g	<1 cfu/g	<1 cfu/g	<1 cfu/g	<1 cfu/g	<1 cfu/g
	TYMC: ≤10^2^ cfu/g	<1 cfu/g	<1 cfu/g	<1 cfu/g	<1 cfu/g	<1 cfu/g	<1 cfu/g
	Absence of *E. coli* in 1 g	Absence	Absence	Absence	Absence	Absence	Absence
	Absence of *Salmonella* in 5 g	Absence	Absence	Absence	Absence	Absence	Absence
	Bile-tolerant Gram-negative bacteria: ≤10^2^ cfu/g	<10 cfu/g	<10 cfu/g	<10 cfu/g	<10 cfu/g	<10 cfu/g	<10 cfu/g

LOD—level of detail; TAMC—total aerobic microbial count; TYMC—total yeast and mold count.

**Table 3:** Physicochemical characteristics of suppositories.

Formulation Code	Uniformity of Mass (g, n = 20)	Disintegration Time (Min, n = 3)	Hardness (kg, n = 10)	Softening Time (Min, n = 3)
CB_MCT_25	2.097 ± 0.009	6.02 ± 0.11	3.07 ± 0.12	4.92 ± 0.19
W_MCT_25	2.102 ± 0.006	9.20 ± 1.09	4.40 ± 0.51	8.17 ± 0.29
CB_MCT_50	2.054 ± 0.009	4.84 ± 0.18	2.50 ± 0.00	4.40 ± 0.13
W_MCT_50	2.095 ± 0.012	7.86 ± 0.91	2.60 ± 0.08	5.94 ± 0.35
CB_EXT_25	2.038 ± 0.004	5.96 ± 0.66	2.47 ± 0.20	5.62 ± 1.47
W_EXT_25	2.036 ± 0.008	8.22 ± 0.18	4.17 ± 0.37	9.50 ± 0.01
CB_EXT_50	2.078 ± 0.014	4.12 ± 0.34	1.63 ± 0.12	3.49 ± 0.01
W_EXT_50	2.095 ± 0.017	7.34± 0.12	2.70 ± 0.08	6.22 ± 0.20

**Table 4:** ∆-9-THC, CBD, and CBN content in suppositories.

Formulation Code	∆-9-THC Content Uniformity (mg, n = 3)	% of Declared ∆-9-THC Content	CBD Content Uniformity (mg, n = 3)	CBN Content Uniformity (mg, n = 3)
CB_EXT_25	22.47 ± 0.712	89.90	0.670 ± 0.004	0.096 ± 0.025
W_EXT_25	20.76 ± 0.082	83.04	0.623 ± 0.037	0.076 ± 0.022
CB_EXT_50	42.94 ± 0.892	85.87	1.141 ± 0.027	0.155 ± 0.048
W_EXT_50	49.38 ± 0.802	84.96	1.308 ± 0.015	0.151 ± 0.035

**Table 5:** Compositions of suppository formulations for series of 10 suppositories.

Formulation Code	Cocoa Butter (CB)/ Witepsol^®^ H15 (W) [g]	MCT Oil [g]	*Cannabis extractum normatum* Based on MCT Oil [g]/∆-9-THC Content [g]	Conditions of Molding Method (rpm Level/Time/Temperature)
CB_MCT_25	22.812	3.0	-	unguator at level 9/8 min 30 s, then at level 0/1 min after oil added
W_MCT_25	23.292	3.0	-	step 1, melting: water bath temperature 70 °C ± 0.5/10 min; step 2, mixing with oil: unguator at level 0/1 min
CB_MCT_50	20.712	6.0	-	unguator at level 9/8 min, then at level 0/1 min after oil added
W_MCT_50	21.192	6.0	-	step 1, melting: water bath temperature 70 °C ± 0.5/10 min; step 2, mixing with oil: unguator at level 0/1 min
CB_EXT_25	22.812	-	3.0/0.025	unguator at level 9/8 min 30 sec, then at level 0/10 s after oil extract added
W_EXT_25	23.292	-	3.0/0.025	step 1, melting: water bath temperature 70 °C ± 0.5/10 min; step 2, mixing with oil extract: unguator at level 0/10 s
CB_EXT_50	20.712	-	6.0/0.05	unguator at level 9/8 min, then at level 0/10 s after oil extract added
W_EXT_50	21.192	-	6.0/0.05	step 1, melting: water bath temperature 70 °C ± 0.5/10 min; step 2, mixing with oil: unguator at level 0/10 s

- absence in the formulation.

**Table 6:** The HPLC gradient mode of the mobile phase.

Time/Min	Mobile Phase A Phosphoric Acid Aqueous Solution (8.64 g/L)	Mobile Phase B Acetonitrile	Elution Mode
0–16	36% → 18%	64% → 82%	Linear gradient
16–17	18% → 36%	82% → 64%	Linear gradient
17–20	36%	64%	Isocratic

## Data Availability

All data are available from the corresponding author. Requests may be sent to the corresponding author.
